# Alterations of natural killer cells activatory molecules phenotype and function in mothers of ASD children: a pilot study

**DOI:** 10.3389/fimmu.2023.1190925

**Published:** 2023-07-20

**Authors:** Marina Saresella, Ivana Marventano, Federica Piancone, Elisabetta Bolognesi, Ambra Hernis, Milena Zanzottera, Francesca La Rosa, Cristina Agliardi, Stefano Giraldo, Matteo Chiappedi, Franca Rosa Guerini, Mario Clerici

**Affiliations:** ^1^ IRCCS Fondazione Don Carlo Gnocchi, Milan, Italy; ^2^ Child Neuropsychiatry Unit, IRCCS Mondino Foundation, Pavia, Italy; ^3^ Department of Pathophysiology and Transplantation, University of Milan, Milan, Italy

**Keywords:** NK, KIR, HLA-G, cytolysis, cytokines, autism endophenotype

## Abstract

**Introduction:**

Autism spectrum disorder (ASD) is accompanied by complex immune alterations and inflammation, and the possible role played by Natural Killer (NK) in such alterations is only barely understood.

**Methods:**

To address this question we analysed activating and inhibitory NK receptors, as well as NK cells phenotype and function in a group of mothers of children who developed ASD (ASD-MO; N=24) comparing results to those obtained in mothers of healthy children who did not develop (HC-MO; N=25).

**Results:**

Results showed that in ASD-MO compared to HC-MO: 1) NK cells expressing the inhibitory receptor ILT2 are significantly decreased; 2) the activating HLA-G14bp+ polymorphism is more frequently observed and is correlated with the decrease of ILT2-expressing cells; 3) the CD56^bright^ and CD56^dim^ NK subsets are increased; 4) IFNγ and TNF production is reduced; and 5) perforin- and granzymes-releasing NK cells are increased even in unstimulated conditions and could not be upregulated by mitogenic stimulation.

**Discussion:**

Results herein reinforce the hypothesis that ASD relatives present traits similar to, but not as severe as the defining features of ASD (Autism endophenotype) and identify a role for NK cells impairment in generating the inflammatory milieu that is observed in ASD.

## Introduction

Ample evidence indicates that immune system activation and chronic neuro-inflammation are present in autistic spectrum disorder (ASD) (1–4). Thus: 1) autoptic evidence of microglia and astrocytes activation is common in ASD; 2) the production of proinflammatory cytokines has been repeatedly shown to characterize ASD children; and 3) multiple inflammasome complexes are abnormally activated in these children (1, 2, 5–10). In this context, NK cells play a pivotal and still only barely understood role. Hence, NK cells of ASD children spontaneously produce higher levels of perforin, granzyme B, and IFN-γ (11), but they are unable to increase the production of these effector molecules upon stimulation, thus having a reduced ability to lyse targets (11, 12). More recent results focusing on the CD57+/CD3− terminally differentiated subset of NK, which is characterized by high cytolytic ability, showed that these cells are significantly reduced in ASD children compared to both age-matched and adult controls.

NK cells activity is finely regulated by a complex balance between activating and inhibitory receptors. Amongst such receptors, killer cell immunoglobulin (Ig)-like receptors (KIRs) and leukocyte immunoglobulin-like receptor subfamily B with TIM domains, member 1 (LILRB1 or ILT2) play a pivotal role by reducing or increasing NK cells activation, respectively, by binding specific HLA ligands on target cells (13–16). Both classical and non-classical HLA molecules, in particular, can be bound by ILT2. This molecule was shown to bind HLA-G, a pivotal molecular modulator of NK cells activity which is responsible for the willingness of pregnant mothers to tolerate fetal tissues, with a significant higher affinity than to classical HLA molecules. Notably, ILT2 also effectively competes with CD8 for MHC-I binding, reinforcing its role as a down-regulator of cell-mediated immune effector mechanism (17). HLA-G exerts its functions through interactions with at least three KIRs inhibitory receptors on NK cells: the already mentioned ILT2, ILT4, and KIR2DL4 (18). Finally, HLA-G expression, and as a consequence NK activation, was shown to be modulated by an insertion/deletion (ins/del) polymorphism of 14bp in the 3’UTR regulatory region.

Recent results showed that: 1) activating KIR/HLA complexes are prevalent in mothers of children who will develop ASD; and 2) particular isoforms of HLA-G are preferentially expressed in ASD children and their mothers (19, 20). These observations support the hypothesis that NK cells could be abnormally activated during fetal development (20, 21). In this scenario, the generation of a chronic inflammatory condition that would persist throughout pregnancy (maternal immune activation or MIA) and/or could affect early postnatal development would be observed (21). Notably, if this prediction is correct, a situation of immune activation would be observed even in healthy sibling of ASD, within the frame of an “autism endophenotype”; this is exactly what has been described (8).

In order to further examine the immune correlates of autism endophenotype we evaluated NK cells in mothers of children who developed ASD, focusing on: 1) the expression of the activating 2DS1, 2DS2, 2DS4, and 2DL4, and of the inhibitory 2DL1, ILT2, and ILT4 receptors, 2) the prevalence and distributions of the HLA-G14bp ins/del polymorphisms; and 3) NK subsets and their function. Results obtained in ASD mothers were compared to those of multiparous women with no familiarity for autism and whose children did not develop ASD. Results confirmed that a complex impairment of NK cells activation/inhibitory signals as well as a skewing in NK cells subpopulations and function are present in mothers of ASD children.

## Materials and methods

### Patients and controls

Twenty-four mothers (mean age: 39.0 ± 7.8) were recruited by the Child and Adolescent Neuropsychiatry Unit of IRCCS Don Carlo Gnocchi Foundation, the ASST S. Paolo and S. Carlo Hospital in Milano, and the C. Mondino National Neurological Institute in Pavia. The inclusion criterium for the mothers was that ASD had been diagnosed in at least one of their children

A second group included twenty-five mothers (mean age: 42.9 ± 9.2) who were recruited among the female staff at IRCCS Fondazione Don Gnocchi (HC-MO). The inclusion criteria, in this case, were to be multiparous women whose children were healthy and without suspected or confirmed diagnosis of Neurodevelopmental Disorders such as Intellectual Disabilities, ASD, Communication Disorders, Attention Deficit/Hyperactivity Disorder, Specific Learning Disorder, or Motor Disorder and to be age-matched with ASD mothers (ASD-MO).

History of spontaneous miscarriage, immunological or endocrinological diseases, and any familiarity with ASD was considered exclusion criteria for the HC-MO.

To avoid the influence of pollens and minimize that of viral and bacterial infection, all samples were collected in summer 2019; additionally, all samples were collected at the same time of the day (early morning). Informed consent was obtained from all the individuals prior to inclusion in the study. The study was conducted according to the guidelines of the Declaration of Helsinki and was approved by the institutional review board of the Don Carlo Gnocchi ONLUS Foundation, Milan.

### Blood sample collection

Whole blood was collected in vacutainer tubes containing ethylenediamine tetra-acetic acid (EDTA) (Becton Dickinson & Co., Rutherford, NJ). Cell counts were performed using the XN-1000 Sysmex hematology analyzer (Dasit Group Italy) and viability was evaluated by the automated cell counter ADAM-MC (Digital Bio, NanoEnTek Inc., Korea), after red cell lysis. Peripheral Blood Mononuclear cells (PBMC) were separated on lymphocyte separation medium (Organon Teknika Corp., Durham, NC) and washed twice in PBS. The PBMCs (10x10^6^) were resuspended in 1 ml of Human Serum AB (Euroclone, Milan, Italy) (Pan Biotech, Aidenbach, Germany) with 10% of DMSO (Sigma – Aldrich, St. Louis Missouri, USA) and stored at −140°C until testing. The cryopreserved PBMC were thawing at 37°C in a water bath, then the cells were washed twice in PBS and resuspended in warm complete RPMI supplemented with 10% of Human Serum AB (Euroclone, Milan-Italy) and antibiotics (Invitrogen, Ltd., Paisley, UK) for the functional assay. The viable leukocytes and count were determined as above.

### *In vitro* PBMC stimulation

PBMCs were resuspended at 2 million/ml in complete RPMI medium. Cells were incubated overnight at 37 °C in a CO_2_ incubator. The next morning, cells were resuspended and 2 ml of cell suspension was seeded in wells of a 12-well tissue culture plate. Samples were performed in duplicate and PBMC were either cultured in medium alone (unstimulated) or stimulated with Staphylococcal enterotoxin B (SEB) (Merck KGaA, Darmstadt, D) plus anti-CD28 antibody (clone CD28.2, IgG1 mouse) (Beckman-Coulter Brea, CA, USA) (1 μg/ml).

Brefeldin A (Sigma)(10 μg/mL) was added to the cultures for cytometric analysis. Cells were incubated at 37 °C in a CO_2_ incubator and after 24 hours were collected and prepared for flow-cytometry analysis.

### Immunofluorescent staining and analysis by flow-cytometry

**Figure 1:**
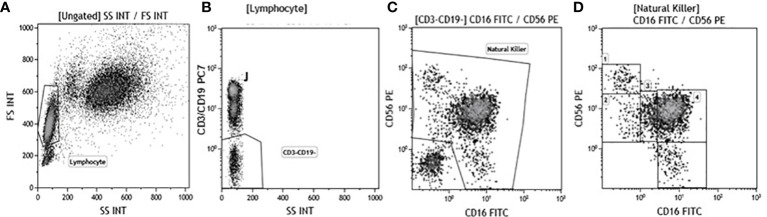
Gate strategy used to identify NK cell subsets. **(A)** Lymphocyte selected by forward (FS) and side scatter (SS) properties (Gate Lymphocyte). **(B)** The CD3+CD19+ *vs.* the SS dot plot allows the discrimination of T and B lymphocytes (Gate J); the remaining double negative cells (Gate CD3-CD19-) were analyzed within a CD56 *vs.* CD16 dot plot **(C)** leading to the identification of NK cells (Gate Natural Killer). **(D)** NK subsets were defined in the CD56 *vs* CD16 dot plot as: CD56^bright^CD16− (1), CD56^dim^CD16− (2), CD56^dim^CD16^dim^(3), CD56^dim^CD16^bright^ (4), and CD56−CD16^bright^ (5).

Immunophenotypic analyses were performed on 600 μl of EDTA peripheral blood incubated for 30 minutes at 4°C with fluorochrome-labeled monoclonal antibodies. Erythrocyte lysis was obtained with the Immuno-Prep Epics Kit and Q-Prep Work Station (Beckman-Coulter Brea, CA, USA). To analyse NK subpopulations 20.000 events were acquired and gated on Forward and Side scatter properties for lymphocytes, and on CD3-CD19-CD14- and Side scatter properties to exclude T and B lymphocytes as well as monocytes. The remaining triple-negative cells were analyzed in a CD56 vs. CD16 dot plot to identify the following natural killer (NK) cell subsets: CD56^bright^/CD16−, CD56^dim^/CD16−, CD56^dim^/CD16^bright^, CD56^dim^/CD16^dim^, and CD56−/CD16^bright^ (**Figure 1**). Analysis of KIR receptors expression was performed on NK subsets. NK functionality (degranulation and production of IFN-γ and/or TNF) was measured using an intracellular cytokine staining assay. Briefly, unstimulated and stimulated PBMC were washed in PBS, split in two tubes, and stained with anti-CD3, -CD19,-CD14-CD56,-CD16 and –CD107 mAb for 30 minutes at 4°C in dark. PBMC were then washed and the intracellular staining of IFNγ, TNF, perforin and granzymes was obtained using the FIX & PERM kit (Invitrogen-Caltag Lab Carlsbad, CA, USA). To identify NK cells producing cytokines or perforin and granzyme, 20.000 events were acquired and gated on Forward (FSC) and Side scatter (SSC) properties for lymphocytes and on CD3-CD19-CD14- and Side scatter properties to exclude T and B lymphocytes as well as monocytes. The remaining triple-negative cells were analyzed in a CD56 vs. CD16 dot plot to identify NK. Cytokine-producing NK cells were selected in the TNF vs. IFNγ dot plot; granzymes and perforin producing NK cells were identified in the granzyme vs. CD107 and in the perforin vs. CD107 dot plot. Analyses were performed using a Beckman-Coulter GALLIOS flow cytometer equipped with a 22 mW Blue Solid-State Diode laser operating at 488 nm and with a 25 mW Red Solid State Diode laser operating at 638 nm and interfaced with Kaluza analysis software. Flow cytometry compensation was performed using the fluorescence minus one (FMO) control approach. Briefly all antibody conjugates in the experiment are included except the one that is controlled for. The FMO measures the spread of fluorescence from the other staining parameters into the channel of interest, determining the threshold for positive staining.

### Monoclonal antibodies

The following mAbs were used: anti- CD3 phycoerythrin-cyanine 7 (PE -Cy7) (Mouse IgG1, Clone: UCHT1) (Beckman-Coulter); anti- CD19 PC-7 or FITC(Mouse IgG1, Clone: J3-119) (Beckman-Coulter); anti- CD14 PC- 7 (IgG2a Mouse, clone: RMO52)(Beckman-Coulter); anti-CD16 PE or PE-Cy5 or Alexa-750 (Mouse IgG1, Clone: 3G8) (Beckman Coulter); anti-CD56 PE or PE-Cy5.5 or PC-7 (Mouse IgG1, Clone: N901 (NKH-1) (Beckman Coulter); CD107a (LAMP-1) FITC (Mouse IgG1clone (H4A3) Ebioscience-Thermofisher, Waltham Massachusetts); anti-Human KIR2DL1/CD158a FITC (Mouse IgG1, Clone: 143211), (R&D Systems, Minneapolis, MN, USA), anti-Human KIR2DS4/CD158i Allophycocyanin (APC) (Mouse IgG2a, Clone: 179315) (R&D Systems); anti-Human ILT2/CD85j APC (Mouse IgG1, Clone: 292305) (R&D Systems); anti-Human KIR2DS1/CD158h Alexa Fluor 700 (Rabbit IgG, Clone: 1127B) **(**R&D Systems); anti-KIR2DS2/CD158b Polyclonal Antibody FITC (Rabbit IgG aa39-65) (LSBio, Seattle WA USA); anti-TNF−FITC or PE (MP9-20A4), anti-IFNγ−PE or FITC (B27) (Invitrogen-Caltag Lab Carlsbad, CA, USA), anti-perforin APC (δG9)(m IgG2b), and anti-granzyme-PE (GB11)(m IgG1)(Becton-Dickinson Biosciences, San Jose CA, USA)

### KIR genotyping

Genomic DNA was isolated from peripheral blood by phenol-chloroform extraction using standard procedures. Molecular KIR genotyping of the activating KIR 2DS1, 2DS2, 2DS4, and the inhibitory 2DL1 and their ligands HLA-C1/C2 was performed by PCR on genomic DNA using sequence-specific primers (SSP) according to the manufacturer’s instructions (BAG- Lich, Germany, Astra Formedic, Milan Italy) as previously described (19, 21). ILT2 rs1061680 and KIR2DL4 rs11410751 polymorphisms were defined using the Allelic discrimination TaqMan SNP Genotyping Assay (Thermo Fisher Scientific) on a CFX Real-Time PCR System (Bio-Rad Laboratories, Inc.). Since no TaqMan assay is available for the 10A/9A insertion/deletion in the 9620 position (rs11410751) of the KIR2DL4 gene, the rs649216:T>C was analyzed. The two SNPs are in complete linkage disequilibrium: the T allele of the rs649216 corresponded to the 9A allele of the rs11410751 that encodes for the soluble form, while rs649216:C corresponded to the variant with the 10A allele that determines the membrane-bound receptor (22). The TaqMan SNP Genotyping Assay utilized were C:_9491145_10 (rs1061680) and C_165450601_10 (rs649216).

### Statistical analysis

Data obtained from all of the individuals in the study were included in the final analyses and were analyzed with appropriate statistical methods.

The normality of the distribution of continuous variables was evaluated using the Kolmogorov–Smirnov test. Quantitative data were defined normally or not normally distributed (Shapiro-Wilk test) and were therefore summarized as mean and standard deviation or median and interquartile range (IQR; 25th and 75th percentiles) respectively. Comparisons between groups were performed using the two-tailed Mann-Whitney test for independent samples. Chi-square analysis was used both to verify that populations were in Hardy-Weinberg Equilibrium and to evaluate differences of categorical variables between groups. Two-sided p values after Bonferroni’s correction (pc) for the degree of freedom (df) were calculated and the significance threshold was set at p < 0.05. Multivariate analysis was applied to evaluate the different NK subsets and their activating or inhibitory receptor expression in ASD-MO, and HC-MO considering age as a covariate. Data analysis was performed with the MedCalc statistical package (MedCalc Software bvba, Mariakerke, Belgium) and SPSS 25.0 for Windows.

## Results

### Genetic polymorphisms distribution

Molecular distribution of the activating (KIR 2DS1-C2, 2DS2-C1, 2DS4) and the inhibitory [2DL1-C2, 2DL4 RS649216(10A/9A), ILT2 RS1061680(C/T)] receptors and their ligands: HLA-C1/C2, as well as that of the HLA-G14bp ins/del (+/-) genotypes is presented in **Supplementary Table 1**.

The genotype distribution of all receptors resulted in Hardy Weinberg equilibrium in ASD-MO and HC-MO. A higher prevalence of the HLA-G14bp+/14b+ homozygous genotype, which is functionally known to down-regulate NK cells activation, was observed in ASD-MO (38%) compared to HC-MO (16%); this difference did not reach statistical significance.

### Genetic polymorphisms and NK cells receptors expression

**Figure 2:**
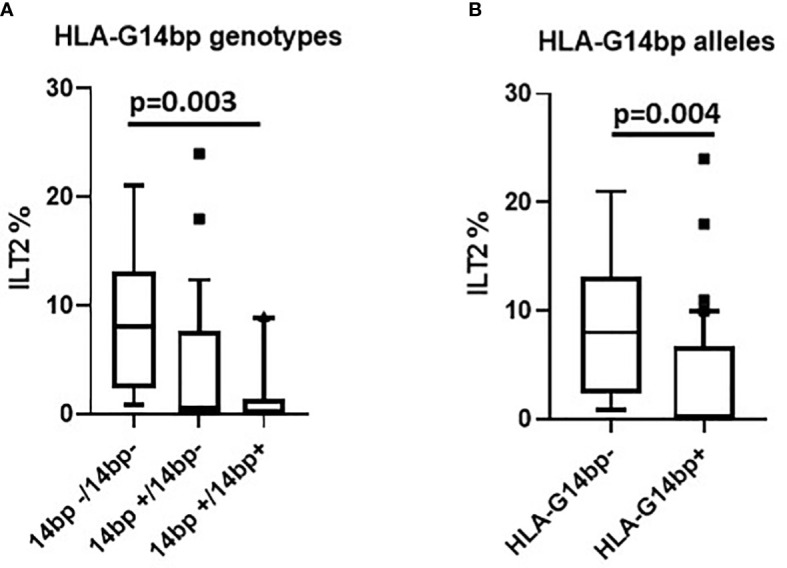
ILT2 inhibitory immunophenotype/HLA-G14bp+/bp- polymorphism: ILT2-expressing NK cells (%) in relationship with the HLA-G14bp+/14bp- genotype in the overall group of individuals analyzed **(A)**. ILT2-expressing NK cells (%) in relationship with minor 14bp+ (14bp+/14bp+, 14bp+/4bp-) vs. major allele 14bp- (14bp-/14bp-) distribution in the overall group of individuals analysed **(B)**. Boxes stretch from the 10th to the 90th percentile. Lines across the boxes indicate the median values. Lines stretching from the boxes indicate extreme values. Statistical significance is shown.

Possible correlations between genotype distribution and the frequency of NK cells expressing different inhibitory and activating receptors were analyzed next. Results showed that the percentage of NK cells expressing ILT2, one of the most important receptors down-regulating NK cells activation, was significantly correlated with the HLA-G 14bp genotype distribution in the overall analyses (p=0.005). Pairwise analysis evidenced that this was specifically due to the comparison between subjects carrying the HLA-G14bp+/14bp+, in whom the percentage of ILT2-expressing NK cells was reduced, and those carrying the HLA-G14bp-/14bp- homozygous genotype, in whom ILT2-expressing NK cells were significantly increased (p=0.003) (**Figure 2A**). These skewing was confirmed to be related to the more frequent presence of the minor HLA-G14bp+ allele both as homozygous (14bp+/14bp+) and as heterozygous (14bp+/14bp-) genotypes in comparison to the HLA-G14bp- (14bp-/14bp-) (p=0.004) genotypes (**Figure 2B**).

**Figure 3:**
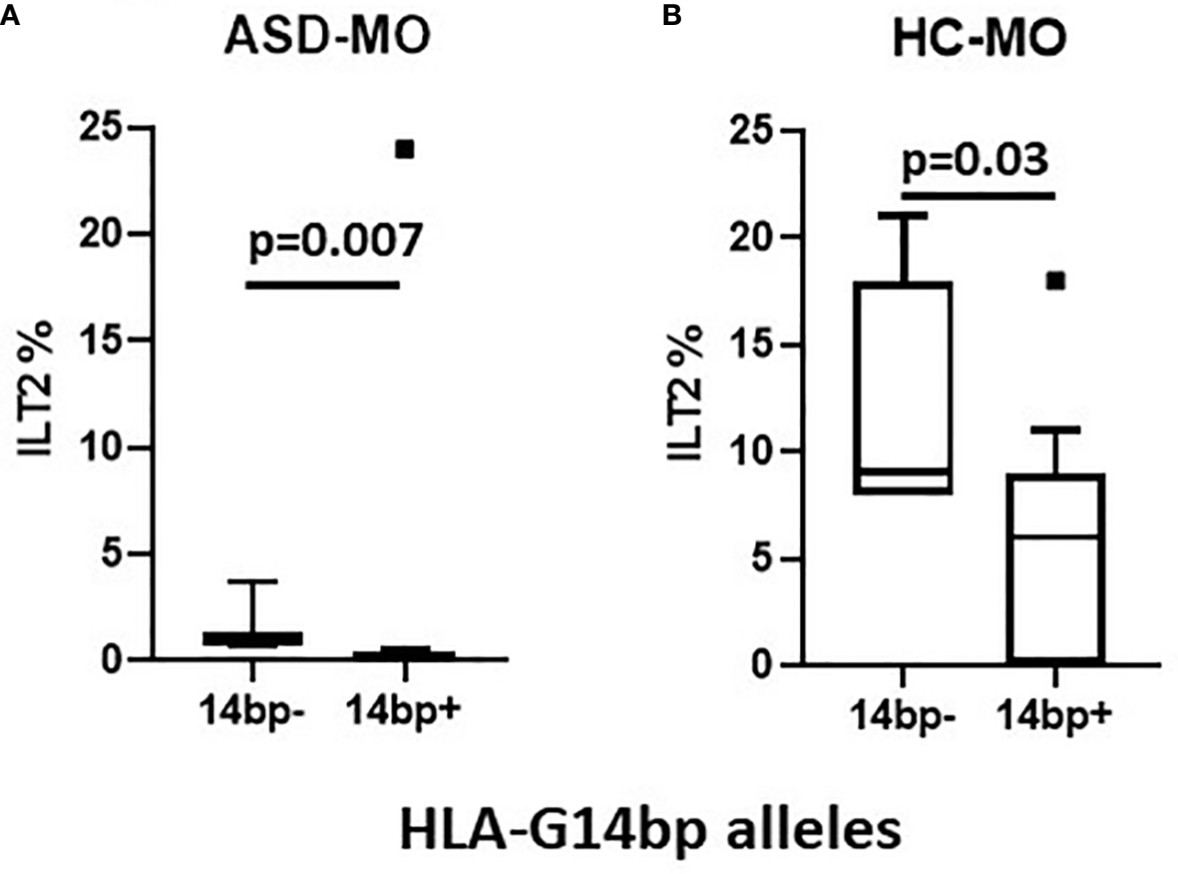
ILT2 inhibitory immunophenotype/HLA-G14bp+/bp- polymorphism: ILT2-expressing NK cells (%) in relationship with minor 14bp+ (14bp+/14bp+, 14bp+/4bp) vs. major allele 14bp- (14bp-/14bp-) distribution in mothers of children who developed (ASD-MO) **(A)**, and mothers of healthy children (HC-MO) **(B)**. Boxes stretch from the 10th to the 90th percentile. Lines across the boxes indicate the median values. Lines stretching from the boxes indicate extreme values. Statistical significance is shown.

The observation that the 14bp+ phenotype associates with a reduced percentage of ILT2-expressing NK cells was confirmed when this parameter was analyzed separately in ASD-MO (p=0.007) and HC-MO (p=0.03) (**Figures 3A, B** respectively).

### NK subsets in peripheral blood

To verify the presence of possible phenotypic skewing in the distribution of different NK cell subpopulations the following NK cell subsets were analyzed next in peripheral blood of all the individuals enrolled in the study: 1) CD56^bright^ 2) CD56^dim^, 3) CD56^dim^/CD16^bright^, 4) CD56^dim^/CD16^dim^, and 5) CD16^bright^. The expression of the activating receptors KIR2DS1, KIR2DS2, KIR2DS4, as well as that of the inhibitory receptors KIR2DL1 and ILT2 was also analyzed.

**Figure 4:**
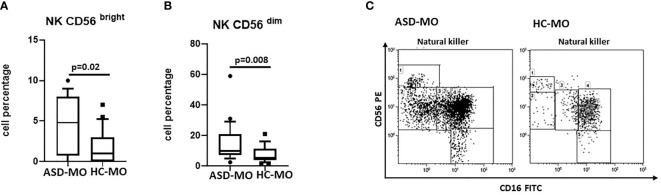
NK subsets. CD56^bright^ **(A)** and CD56^dim^ **(B)** NK cell subsets (%) in mothers of children who developed (ASD-MO), and mothers of healthy children (HC-MO) Boxes stretch from the 10th to the 90th percentile. Lines across the boxes indicate the median values. Lines stretching from the boxes indicate extreme values. Statistical significance is shown. Representative dot plots of CD56^bright^ *vs* CD56^dim^ NK cell subsets **(C)** obtained from ASD-MO or HC-MO.

Results showed that CD56^bright^ and CD56^dim^ NK cells were significantly increased in ASD-MO compared to HC-MO (p=0.02 and p=0.008 respectively) (**Figure 4**). These NK cell subsets are characterized by a potent production of proinflammatory cytokines (CD56^bright^) and by an enhanced cytolitic ability (CD56^dim^). Taken overall, thus, these results support the observation that immune responses are activated in ASD mothers.

**Figure 5:**
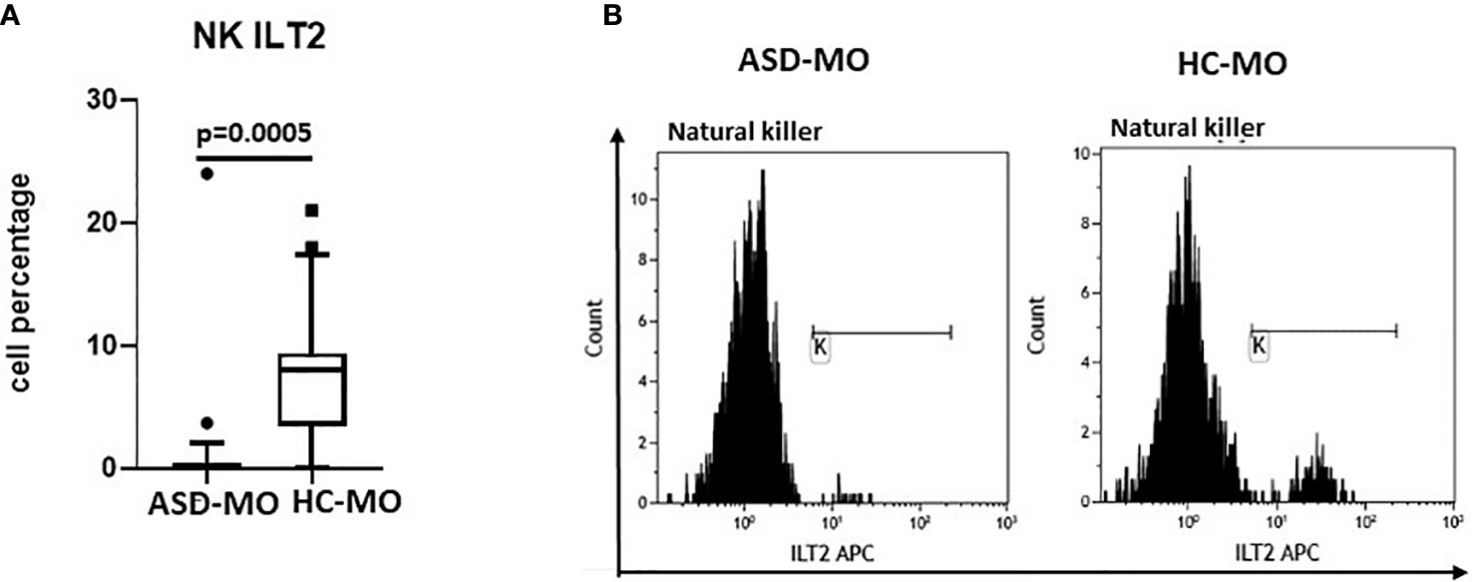
ILT2 inhibitory immunophenotype. NK cells expressing the ILT2 inhibitory receptor (%) **(A)** in mothers of children who developed (ASD-MO) and mothers of healthy children (HC-MO). Boxes stretch from the 10th to the 90th percentile. Lines across the boxes indicate the median values. Lines stretching from the boxes indicate extreme values. Statistical significance is shown. Representative histograms of ILT2-expressing NK **(B)** obtained from ASD-MO or HC-MO.

NK expressing activating and inhibitory receptor were analyzed next. Whereas no differences were observed in the expression of KIR2DS1, KIR2DS2, KIR2DS4 and KIR2DL1, results confirmed that an alteration of ILT2-expressing NK cells can be detected in ASD mothers. Thus, the percentage of NK cells expressing ILT2 was significantly reduced in ASD-MO compared to HC-MO (p=0.0005) (**Figure 5**).

### Cytokine production by NK cells

**Figure 6:**
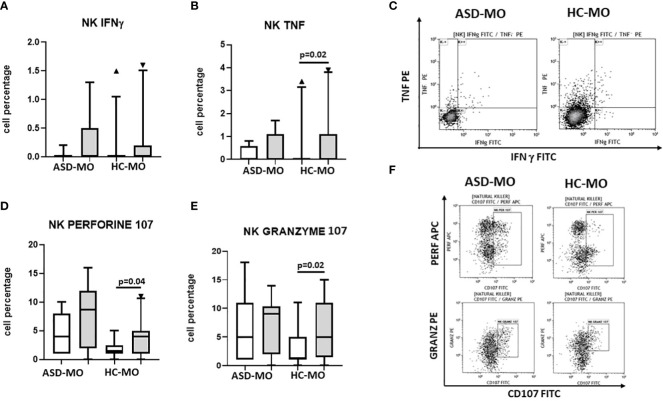
NK functionality. INFγ **(A)**, TNF **(B)**, CD107a+/perforin+ **(C)**, and CD107a+/granzymes+ **(D)** NK cells. Cells were either unstimulated (white box) or were stimulated with SEB+ anti-CD28 (grey box). Results obtained in mothers of children who developed (ASD-MO) and mothers of healthy children (HC-MO) are shown. Boxes stretch from the 10th to the 90th percentile. Lines across the boxes indicate the median values. Lines stretching from the boxes indicate extreme values. Statistical significance is shown. Representative dot plots of TNF vs. IFN-γ producing NK cells **(E)** or of granzyme *vs.* CD107 and of perforin *vs.* CD107 **(F)** obtained from ASD-MO or HC-MO. ▲, represent outside values.

NK cells were functionally analysed by measuring IFNγ and TNF production by cells that had been cultured in medium alone or had been stimulated with SEB plus anti-CD28. Results indicated that IFNγ and production was barely detectable in unstimulated NK cells of ASD-MO and HC-MO and was increased by stimulation in both groups. Notably, in HC-MO TNF production upon antigenic stimulation was significantly higher compared to what was observed in unstimulated cells (p=0.02) (**Figures 6A-C**).

### Perforin- and granzyme-containing and CD107a-expressing NK cells

Secretory lysosomes of NK cells contain perforin and granzymes; these proteins mediate NK-cell cytotoxicity, as their release results in the induction of target-cell lysis and apoptosis. Lysosome-associated membrane protein (LAMP) 1/CD107a is used as a marker for NK-cell degranulation and cytotoxicity. NK population that expressed CD107 and either granzyme or perforin were analyzed next in cells that had been cultured in medium alone or had been stimulated with SEB plus anti-CD28.

Results showed that both CD107a+/perforin+ and CD107a+/granzyme+ NK cells were increased in unstimulated conditions in ASD-MO compared to HC-MO. Notably, stimulation increased both NK cell populations in HC-MO (CD107a+/perforin+: p=0.04 and CD107a+/granzyme+: p=0.02), but did not have any effect in ASD-MO, in whom probably both CD107a+/perforin+ and CD107a+/granzyme+ cells were already maxed out in unstimulated conditions (**Figures 6C-F**).

## Discussion

A subtle and complex impairment of cell-mediated effector mechanisms is suggested to be present in ASD. Thus, Natural Killer (NK) cells activity is reduced in ASD children (11), in whom lower quantities of CD8+ effector memory T lymphocytes are also observed (8). These alterations were suggested to result in a reduced ability to handle infectious agents and/or vaccine antigens, and a minor efficacy of anti-tumoral immune defences. On the other hand, a number of results showed that activating KIR/HLA complexes and specific HLA-G polymorphisms associated with the activation of NK cells, are prevalent in ASD children and their mothers (ASD-MO) (19, 21). In ASD-MO this would result in abnormal NK activation and contribute to the generation of prenatal maternal immune activation (MIA), a risk factor for the subsequent development of ASD. Notably, the presence of immune alterations in mothers of children who develop autism would support the autism endophenotype hypothesis, stating that traits similar to, but not as severe as those of ASD, can be detected in relatives of ASD children (8). In the attempt to shed further light on ASD-associated immune profiles, we analyzed HLA/KIR receptors, HLA-G genotypes, NK subsets, and NK function in ASD-MO comparing results to those obtained in mothers of children who did not develop neurodevelopmental disorders.

Results herein confirmed that a genetic background associated with NK cell activation is present in ASD-MO. Thus: the HLA-G14bp+/14b+ homozygote genotype, which is functionally known to down-regulate NK cells activation, was more frequent in ASD -MO, in whom NK cells that express ILT2, one of the most important receptors that down-regulates NK cells activation, were significantly reduced. Notably, in ASD-MO, two populations of NK cells, CD56 ^bright^, potent producers of inflammatory cytokines (23), and CD56^dim^, cells with high cytolytic function (24), were significantly increased. Finally, the production of proinflammatory cytokines was reduced whereas that NK-associated proteins that mediate apoptosis and lysis was increased, even in unstimulated conditions, in ASD-MO.

The observation that the HLA-G14bp+ allele and the HLA-G14bp+/14bp+ genotype were more frequently present in ASD–MO, even though not reaching the statistical significance probably due to the limited cohort studied, confirms previously published results stemming from analyses performed in larger cohorts (19) and support the idea that this genetic profile could play a major role in MIA.

A direct correlation between the expression of HLA-G on immune cells and that of ILT2 on NK was suggested (25). In particular, HLA- G14bp+ insertion/deletion polymorphism in the 3’ UTR was found to be associated with stability and splicing pattern of HLA-G mRNA isoforms, with lower soluble HLA-G expression in plasma, and with ILT2 expression (26). The mechanisms by which HLA-G induces transcription changes in immune cells are unknown but it was suggested that HLA-G mediates such changes *via* its known ligands ILT2, ILT4, and KIR2DL4. Thus, two possible mechanisms are postulated: 1) cross-linking of inhibitory receptors would have a positive feedback on their expression; and 2) the presence of other HLA-G receptors on APC, T lymphocytes, and NK cells would stimulate the transcription of inhibitory receptors, resulting in their upregulation (25). If this is the case, the reduced percentages of ILT2-expressing cells observed in ASD-MO could be at least partially justified by the direct effect of HLA-G14bp+ insertion/deletion polymorphism. A precise definition of the effect of the HLA-G-ILT2 molecular interaction is outside the scope of our results. The observation that ASD-MO were more frequently carrying HLA-G14bp+ and were likely to have reduced amount of circulating ILT2-expressing NK cells could be of importance as it may be a relevant contributor to the immune activation seen in these individuals.

ILT2 interaction with HLA-G was shown to also influence NK cell functionality as it efficiently inhibits intracellular calcium mobilization and IFNγ production, leading to the inhibition of NK-cell functions (27). Our results showed that the production of proinflammatory cytokines by NK cells, including IFNγ, was reduced in all conditions in ASD-MO. The increase of CD56^bright^ and CD56^dim^, as well as the ability of NK cells to spontaneously produce higher levels of perforin and granzyme B, without the possibility to further increase the release of these molecules upon stimulation, confirm previous results obtained in ASD children (11, 12, 28, 29), but for the first time expand this observation to their mothers as well.

This is particularly puzzling at the light of the finding that CD56^bright^ NK cells, known to be high producers of proinflammatory cytokines, were augmented in ASD-MO. The observation that ILT2 expression is also reduced in these individuals leads to two possible conclusions: in ASD–MO: 1) CD56^bright^ NK cells are increased but could be functionally impaired; and 2) defective IFNγ production is likely independent of the ILT2/HLA-G interaction. Notably, IFNγ production was only marginally augmented in ASD-MO. These results offer further support to the observation that cellular immunity is impaired in ASD, possibly being maxed out and/or exhausted, and incapable of being modulated by antigens, as has been previously suggested (28).

Interesting alterations were observed in ASD-MO also when NK cells degranulation and the release of lytic and apoptotic molecules were analyzed. Thus, CD56^dim^ NK cells were increased in these women, possibly justifying the observation that degranulating NK cells releasing perforin and granzymes were present even in unstimulated conditions; notably though, degranulation could not be upregulated upon mitogenic stimulation. Also in this case, immune exhaustion could justify these results (30). Alternatively, these data could be explained by the recently proposed ‘discontinuity theory’, according to which the immune system can respond properly to abrupt antigenic stimulation changes but can become tolerant in presence of continuous and minimal stimulations (31). Within this context it is tempting to speculate that the NK cells activation profile stemming from the HLA-G14bp+/ILT2 profile seen in ASD –MO would contribute to such continuous and minimal stimulation.

Older results showed that a skewing of T lymphocyte maturation pathways as well an alteration of cytokine production are present not only in ASD children but in their healthy siblings as well, reinforcing the idea of the existence of an autistic endophenotype (8). Results herein, although stemming from analyses performed in a limited number of individuals and needing to be confirmed, extend the autistic endophenotype spectrum to involve the phenotype and function of NK cells and their receptors, and suggest that such spectrum involves mothers of ASD children as well.

## Data availability statement

The original contributions presented in the study are publicly available. This data can be found here: 10.5281/zenodo.7839882 (https://doi.org/10.5281/zenodo.7839882).

## Ethics statement

The studies involving human participants were reviewed and approved by institutional review board of the Don Carlo Gnocchi ONLUS Foundation, Milan. The patients/participants provided their written informed consent to participate in this study.

## Author contributions

MCl designed research and edited the manuscript; MS and FRG designed and performed analysis and draft the manuscript; MCh collected clinical data; IM, AH, and SG performed immunological experiments and collected immunological data, MZ and CA, performed genetic experiments and collected genetic data; FP, FLR, and EB analyzed results and made figures. All authors contributed to the article and approved the submitted version.
