# Supplementary material for: Alterations of natural killer cells activatory molecules phenotype and function in mothers of ASD children: a pilot study
Source: Front Immunol. 2023 Jul 20;14:1190925. doi: 10.3389/fimmu.2023.1190925 (PMC10398568; doi:10.3389/fimmu.2023.1190925)
Supplement: Supplementary file 1 [file Table_1.docx]

|  | ASD-MO (24) | | HC-MO (25) | |
| --- | --- | --- | --- | --- |
|  | n | % | n | % |
| **KIR-HLA COMPLEX** | |  |  |  |
| 2DS1-C2 | 6 | 25% | 7 | 28% |
| 2DS2-C1 | 12 | 50% | 16 | 64% |
| 2DS4 | 23 | 96% | 25 | 100% |
| 2DL1-C2 | 16 | 67% | 14 | 56% |
|  |  |  |  |  |
| c1/c1 | 8 | 33% | 9 | 36% |
| c1/c2 | 10 | 42% | 11 | 44% |
| c2/c2 | 6 | 25% | 5 | 20% |
|  |  |  |  |  |
| **ILT-2 RS1061680** | |  |  |  |
| CC | 3 | 13% | 1 | 4% |
| CT | 8 | 33% | 13 | 52% |
| TT | 13 | 54% | 11 | 44% |
|  |  |  |  |  |
| **2DL4 RS649216** | |  |  |  |
| CC 10A/10A | 7 | 29% | 6 | 24% |
| CT 10A/9A | 12 | 50% | 14 | 56% |
| TT 9A/9A | 5 | 21% | 5 | 20% |
|  |  |  |  |  |
| **HLA-G14 ins/del** | |  |  |  |
| 14bp+14bp+ | 9 | 38% | 4 | 16% |
| 14bp+14bp- | 12 | 50% | 15 | 60% |
| 14bp-14bp- | 3 | 13% | 6 | 24% |
|  |  |  |  |  |
| 14bp+ | 21 | 87% | 19 | 76% |
| 14bp- | 3 | 13% | 6 | 24% |

Table 1S: Genotype distribution in 24 mothers of ASD children (ASD-MO) and 25 multiparous mothers as controls (HC-MO). All genotype distribution result in HWE. No statistical difference were observed between groups.
